# What Does *That* Mean? Complementizers and Epistemic Authority

**DOI:** 10.1162/opmi_a_00135

**Published:** 2024-03-26

**Authors:** Rebecca Tollan, Bilge Palaz

**Affiliations:** University of Delaware

**Keywords:** complementizers, *that*-mention/omission, epistemic authority, discourse status, corpus survey

## Abstract

A core goal of research in language is to understand the factors that guide choice of linguistic form where more than one option is syntactically well-formed. We discuss one case of optionality that has generated longstanding discussion: the choice of either using or dropping the English complementizer *that* in sentences like *I think (that) the cat followed the dog*. Existing psycholinguistic analyses tie *that*-usage to production pressures associated with sentence planning (Ferreira & Dell, 2000), avoidance of ambiguity (Hawkins, 2004), and relative information density (Jaeger, 2010). Building on observations from cross-linguistic fieldwork, we present a novel proposal in which English *that* can serve to mark a speaker’s “epistemic authority” over the information packaged within the embedded clause; that is, it indicates that the speaker has more knowledge of the embedded proposition compared with their addressee and thus has a perspective that they believe their addressee doesn’t share. Testing this proposal with a forced-choice task and a series of corpus surveys, we find that English *that* is keyed to the use of embedded speaker (first-person) subject pronouns and occurs in sentences containing newsworthy information. Our account of *that*-optionality takes into account why *that* is associated with both (i) a dense information signal and (ii) semantic-pragmatic content, as well as extending to cases of non-optionality in subject/sentence-initial clauses (e.g., *(That) the cat is following the dog, I already know) and fragment answers (e.g., *What do you already know?* *(That) the cat is following the dog), where *that* is required.

## INTRODUCTION

Understanding the range and nature of the factors which influence language users’ choice and interpretation of linguistic form has long been a central goal in the study of human language. That is, wherever a single meaning can be expressed via more than one utterance form, a speaker must draw upon one or more criteria in deciding which of the available forms to use. For example, an event of *following*, involving an agent entity of a *cat*, and a patient entity of a *dog*, may be expressed either via an active verb form, as in (1a), or via a passive verb form, as in (1b). The choice of ultimately saying (1a) or (1b) therefore requires the speaker to make a linguistic decision. Indeed, previous research on the active-passive alternation has revealed that such a decision is governed by a range of factors, including prior exposure (Bock, 1986), information structure (Birner & Ward, 1998), and animacy and/or person of the agent noun (Bresnan et al., 2001; Ferreira, 1994; Gennari & MacDonald, 2009; MacDonald, 2013).

1.  **English active-passive alternation**
  - *The cat is following the dog.*       ACTIVE
  - *The dog is being followed by the cat.*    PASSIVE

The current paper is concerned with the linguistic choice which is triggered by the need to embed a clause (such as 1a) under a verb like *think*, which takes a clausal complement. In this environment, a speaker may either use the overt complementizer *that*, as in (2a) or omit it, as in (2b); both options are grammatically licit.

2.  **English complementizer omission**
  - *I think [**that** the cat is following the dog].*  OVERT COMPLEMENTIZER
  - *I think [the cat is following the dog].*   NO/NULL COMPLEMENTIZER

The alternation in (2) has attracted an extensive body of research over the past several decades. Going back at least to Bolinger (1972), the factors which influence a speaker to ultimately either include or omit the complementizer *that* have generated debate which has spanned several linguistic disciplines, including psycholinguistics (Ferreira & Dell, 2000; Hawkins, 2004; Jaeger, 2010; Rohdenburg, 1996; Roland et al., 2006), syntax (Doherty, 2000; Rizzi, 1990; Weisler, 1980), semantics and pragmatics (Bolinger, 1972; Dor, 2005; Hiroe, 1999; Staps & Rooryck, 2023; Thompson & Mulac, 1991), prosody (Sato & Dobashi, 2016), sociolinguistics (Eckert, 2018; Elsness, 1984; Tagliamonte & Smith, 2005; Tagliamonte et al., 2005), and language change (Conde-Silvestre, 2022). Although there is a certain degree of consensus within some of these subfields as to which factor(s) underpin(s) *that*-usage, these cross-disciplinary observations are rarely related to each other and are not straightforwardly compatible. On the one hand, research in psycholinguistics to date has largely converged on a view in which *that*-mention emerges in contexts where the embedded clause (e.g., *the cat is following the dog* in 2) packages a higher information load (i.e., a greater degree of information which is new to the dialogue) as compared with contexts where *that* is omitted, in which the embedded clause has a less dense information load (Ferreira & Dell, 2000; Jaeger, 2010; Rohdenburg, 1996; Roland et al., 2006). Thus, when information load is high, the complementizer serves as a means of distributing this signal over more words (Jaeger, 2010). On the other hand, researchers in other linguistic subfields like semantics contend that *that* carries a particular component of meaning such as “a certain specific mood” (Hiroe, 1999: 55), which is absent when the complementizer is omitted. These two types of accounts do not overlap in an obvious way: just as psycholinguistic theories do not simultaneously account for observations in other linguistic literature regarding semantic markedness of *that*, semantic theories of *that*-mention do not explain observations in the psycholinguistic literature, where *that* is observed to be associated with a denser information signal (Jaeger, 2010; Levy & Jaeger, 2007). A welcome benefit of psycholinguistic accounts such as Jaeger (2010) and Levy and Jaeger (2007) is that they tie in with observations regarding certain aspects of sentence production planning:For instance, use of a complementizer has been shown to coincide with the need to spend more time planning a sentence (Ferreria & Dell, 2000), which is indicative of a denser information load (Arnold et al., 2003).

However, well-formedness of sentences with *that*-omission is not uniform. Importantly, Dor (2005) identifies three categories of matrix verbs which take clausal complements: Of these three categories, only one—which includes so-called “bridge” verbs such as *think*—comprises verb that are *equally* compatible with and without *that*-omission. Conversely, verbs in the other two categories tend to be less compatible with *that*-omission (i.e., they are preferred with *that*-mention); these categories include manner-of-speaking verbs (e.g., *murmur*, *whisper*), predicates of instrument communication (e.g., *email*, *radio*), emotive predicates (e.g., *be amazed*, *be proud*), and predicates issuing a guarantee or ascertaining a state of world affairs (e.g., *legislate*, *warrant*). Dor (2005) reports some speakers *do* permit *that*-drop for these verbs but testify that they prefer them with *that*-mention. Other speakers, meanwhile, do not permit *that*-drop with these kinds of predicates at all. Furthermore, recent work has examined the role of factivity in acceptability of *that*-omission (Bîlbîie et al., 2023). Factive verbs—verbs which presuppose the truth of a clausal/propositional complement, such as *regret* and *like*—were observed by Bîlbîie et al. (2023) to be less acceptable without *that* as compared to non-factive verbs such as *think* and *imagine*. These sets of observations provide support for a long-held view in linguistic literature that *that* contributes meaning to a multi-clausal construction, carrying “interpretative information” (Staps & Rooryck, 2023: 1169).

The goal of the current paper is to present a new proposal regarding the contribution of *that*-mention to constructions like (2b) which incorporates observations from both research on sentence processing and production (e.g., Ferreira & Dell, 2000; Jaeger, 2010; Rohdenburg, 1996, a.o.) and linguistic accounts that ascribe interpretation to *that* (e.g., Bolinger, 1972; Dor, 2005; Staps & Rooryck, 2023, a.o.). We summarize these two points in (3).

3.  **Summary of existing observations vis-à-vis *that*-mention:**
  - *that*-mention occurs when information load in the embedded clause is high (Ferreira & Dell, 2000; Jaeger, 2010).
  - *that* carries semantic-pragmatic/interpretative content (Bolinger, 1972; Dor, 2005; Staps & Rooryck, 2023).

To give an overview of our proposal, we contend that one of the key factors behind *that*-mention, which has potential to connect psycholinguistic accounts with formal linguistic accounts, is its function in marking “epistemic authority” (Zagzebski, 1996). Discussion of epistemic authority in language has gained ground in recent years, owing to new observations in cross-linguistic fieldwork on languages such as Upper Napo Kichwa (Quechuan; Grzech, 2016, 2020), Murrinhapatha (Australian; Mansfield, 2019), and Jaminjung/Ngaliwurru (Australian; Schultze-Berndt, 2017), but has heretofore not been discussed in connection with English complementizer usage. In this context, epistemic authority concerns the desire, requirement (or impossibility) of the speaker to signal that they have (or want to project themselves as having) greater knowledge surrounding the embedded proposition, compared with their addressee. This grants to the speaker an epistemically superior conversational stance and means that the speaker holds (or believes that they hold) information that is not shared by the addressee. Epistemic authority serves to flag to the addressee that they can expect further information to be forthcoming: in other words, that the Common Ground (Stalnaker, 1974) is being updated. Thus, *that*-mention implores the addressee to pay due attention, thus maximizing the chance for successful communication (Grice, 1975). In (4), we briefly detail how our proposal of *that* as marking epistemic authority accounts for the observations in (3), before elaborating in the forthcoming sections of the paper.

4.  **Under an account in which *that* signals epistemic authority of the speaker:**
  - A speaker conveys more information in embedded clauses with overt *that* in order to compensate for their addressee’s inferior knowledge about the proposition.
  - *that* conveys an asymmetry in the epistemic status speaker by comparison with the addressee, with the speaker holding an epistemic advantage.

In the next section, we provide an overview of two core strands of background literature on *that*-mention which drives the motivation for our account of epistemic authority marking, to follow.

## BACKGROUND: *THAT* AND EPISTEMICITY

This section motivates our grounds for a new proposal which links complementizer form to epistemic authority. We begin with an overview of previous accounts of *that*-optionality for sentences like in (2), repeated in (5).

5.  **English complementizer omission [= (2)]**
  - *I think [**that** the cat is following the dog].*  OVERT COMPLEMENTIZER
  - *I think [the cat is following the dog].*   NO/NULL COMPLEMENTIZER

### Psycholinguistic Accounts of *That*-Optionality

Research in psycholinguistics has largely converged on the view that *that* functions primarily as a means of managing a heavy or complex information load associated with the embedded clause that it introduces (i.e., in 5, “the cat is following the dog”). In terms of sentence processing, Rohdenburg (1996) observed that *that* delays the processing of the complement clause, meaning that *that* usage marks a complement clause of greater complexity than one lacking *that*, an effect which has been further shown to be predictable from contextual information (Roland et al., 2006). In a similar vein, complementizer mention has also been shown to alleviate production disfluencies in speaking (Ferreira & Dell, 2000). This effect, termed as the “Principle of Immediate Mention”, occurs when the embedded clause requires a high degree of planning on the part of the speaker, and so *that*-mention is used as a strategy to allow the speaker more time to plan the upcoming clause. Furthermore, *that*-mention also helps to eliminate parsing ambiguity for the listener by overtly marking the beginning of a complement clause (Bolinger, 1972; Hawkins, 2004). As tested by Trueswell et al. (1994), this means that the subject of the embedded clause cannot be momentarily mis-parsed by the listener as a direct object of the matrix verb. Finally, Jaeger (2010), building on Levy and Jaeger (2007), shows that *that*-mention is sensitive to the distribution of information across an utterance: Information which is *new* to a discourse adds *more* information load to the utterance. Importantly, multi-clausal utterances which contain higher information loads are probabilistically more likely to comprise an overt complementizer as compared with those with relatively lower information loads. Under Jaeger’s (2010) model of “Uniform Information Density”, *that*-mention serves as a means of distributing a higher information load over more lexical tokens, thus making the resulting signal less informationally dense. As Jaeger (2010: 28) contends, “processing mechanisms are the major driving force behind *that*-mentioning—a standard assumption in the psycholinguistic literature, but by no means outside the field.”

However, accounts such as Jaeger (2010) consider a small range of matrix verbs: verbs where a clausal complement is compatible *both* with and without *that* (e.g., *agree*, *believe*, *bet*, *consider*, *decide*, *expect*, *feel*, *figure*, *find*, *guess*, *hear*, *hope*, *imagine*, *know*, *mean*, *notice*, *read*, *realize*, *remember*, *say*, *see*, *show*, *suppose*, *take*, *teach*, *tell*, *thank*, *think*, *understand*, *wish*, *worry*; Jaeger, 2010: Table 1A). These verbs are part of the verb class characterized by Dor (2005) as “Type I verbs”. They comprise (i) speech act predicates (e.g., *say*, *tell*) and (ii) predicates of belief, knowledge, and conjecture (e.g., *feel*, *hope*), and we refer to these as “*that*-optional” verbs. Importantly, however, Dor additionally identifies verbs of “Type II” and “Type III”, wich behave differently from Type I. Type II are predicates include (i) manner-of-speaking verbs (e.g., *murmur*, *whisper*; cf. 4), (ii) predicates of instrument communication (e.g., *email*, *radio*), and (iii) emotive predicates (e.g., *be amazed*, *be proud*). This class of predicates tend to be preferred *with that* than without. Based on a survey of 50 native speakers of English, Dor (2005) finds that, although some speakers *do* permit *that*-drop for these verbs, they consistently testify that they prefer *that*-mention (and often find it difficult to produce clear judgements). Other speakers, meanwhile, do not permit *that*-drop with these predicates at all. We refer to this class of verbs as “*that*-preferred”. Meanwhile, Type III verbs, which we call “*that*-required”, disallow *that*-drop across the board. These include so-called “word-to-world predicates” (e.g., *arrange*, *legislate*), predicates of physical manipulation of text (e.g., *read aloud*, *publish*), and predicates issuing a guarantee (e.g., *warrant*). According to Dor, these predicates share a common characteristic, in that matrix subject of the sentence has acted with the goal of making them true. In total, Dor identifies 70 verbs as being of Types II (*that*-preferred) and III (*that*-required). The fact that many verbs are at best only mildly compatible with *that*-drop necessitates an account of *that* which speaks to both its attested informational distribution in tandem with a meaning-centered contribution. We therefore turn next to discussing literature focused on the interpretative properties of *that*, before considering a line of analysis which holds a connection with observations in psycholinguistic literature.

### Semantic-Pragmatic Accounts of *That*-Mention

Research in semantics and pragmatics literature on clausal complementation has established differences in meaning between sentences with and without *that*. In a survey of articles from newspapers, Underhill (1988) observes that *that* is deleted when the speaker endorses the assertation in the embedded clause (and mentioned when they do not), or when the subject from the embedded clause functions as the discourse topic. Underhill’s (1988) proposal is that that *that*-omission occurs when the embedded clause essentially functions more like a main clause in terms of its discourse status. Thompson and Mulac (1991) subsequently argued that certain matrix verbs which embed *that*-less complement clauses comprise grammaticalized “epistemic parentheticals” (Thompson & Mulac: 316): They function together with their matrix subjects to indicate epistemic modality. Under this account, constructions with *that*-omission such as “I think the dog is following the cat” are not actually true instances of embedding: Rather, the phrase “I think” marks the beliefs of the speaker towards the proposition “The dog is following the cat”, which itself functions as the matrix clause in this instance. Related to the notion of epistemicity, Hiroe (1999: 55) proposed that *that*-mention is associated with sentence mood, noting that “when a certain specific mood is projected […] *that*-deletion is disallowed, and vice versa”. Further work since has characterized this “mood” as pertaining specifically to the attitude of the speaker: Yaguchi (2001) characterizes *that* as indicating a speaker’s vantage point (cf. Thompson & Mulac, 1991), wherein it expresses belief, state-of-knowledge, and/or inference of the speaker based on evidence available to them. Building on Yaguchi (2001) and Dor (2005) contends that *that*-omission entails that the subject of the embedded clause, or some other cognitive agent, has made “an epistemic claim concerning the truth of the proposition denoted by the embedded clause” (Dor, 2005: 347). In other words, *that* has been analyzed as a marker of “epistemicity”—or knowledge state—of either the speaker of the sentence and/or the embedded or matrix subject.

In the linguistics literature, epistemicity has been closely connected to the related category of “evidentiality”; namely, the use of a grammatical device which indicates the speaker’s source of knowledge for the proposition conveyed via the sentence they are uttering (Aikhenvald, 2004; Chafe & Nichols, 1986). An association of *that*-mention with evidence held by the speaker has been noted by Rooryck (2001: 163), who contends that complementizers carry “evidential information”, wherein the speaker expresses that they have certain evidence at their disposal pertaining to the truth of the embedded proposition. If we are to consider the English null/*that* complementizer alternation along similar lines, one might consider whether *that* some form of evidentiality (cf. Rooryck, 2001), which is not available where a speaker uses the null form. English *that*-mention indeed appears to be conditioned by factors related to epistemicity and/or evidentiality. To illustrate this, Bolinger (1972) presents two contrasting exemplar scenarios and contends that they give rise to a marked judgement in suitability of *that*-mention (a contrast revisited and substantiated by Dor, 2005), as in (6). Both scenarios involve a speaker protagonist who receives information pertaining to an event that has reportedly taken place, and an addressee who is a participant in the reported event. Bolinger (1972) and Dor (2005) contend that, in the first scenario, the speaker is more likely to omit the complementizer telling the addressee of the report, whereas in the second scenario, “*that*” is more likely to be present.

6.  **Speaker knowledge driven *that* contrast (Bolinger, 1972; via Dor, 2005: 376–7)**
  - “Consider the following scenario […]: A police officer has just been told that a robbery has been committed at a local gas station. When she gets there, she sees broken glass all over the place. The attendant is ostensibly shaken. The fact that the place has just been robbed is now very obviously part of the common ground. The police officer is likely to say something like […] *They told us you’ve been robbed*.”
  - “Imagine, by contrast, a scenario where the police officer arrives at the gas station and finds no signs of robbery. The attendant seems relaxed and happy. The police officer approaches the attendant and tells him about the robbery report. In this context, the police officer is more likely to say something like […] *They told us **that** you’ve been robbed*.”

Neither of the scenarios in (6a) and (6b) involve any difference as to the type of evidence that is available to the two participants of the speech act, as established via the physical Common Ground (Stalnaker, 1974, 2002): both speaker and addressee are privy to the visual evidence of a robbery having taken place (6a), or not (6b). Instead, we note that what changes between the two scenarios is whether the addressee has knowledge of the robbery report like the speaker does (6a), or not (6b). Consistent with earlier observations by Thompson and Mulac (1991) and Yaguchi (2001), the contrast between *that*-mention and *that*-omission in this example relates to the speaker’s state of knowledge. Importantly however, we observe that the speaker’s knowledge state is not treated here as being self-contained—rather, it is evaluated *relative* to the state of knowledge of the addressee. In (6a), the speaker and addressee both know that a report has been filed, and *that* is omitted in the speaker’s utterance. In (6b), the speaker holds this information, and the addressee is ostensibly unaware of the speaker’s privileged perspective. In this case, *that* is mentioned.

At this point, we draw a parallel with cross-linguistic observations. Recently, Grzech’s (2016, 2020) original fieldwork on Upper Napo Kichwa (Quechuan), has revealed what had previously been characterized as “evidential” markers in this language show similar kind of distribution in terms of speaker vs. addressee knowledge states. Grzech (2020) analyses two such Kichwan enclitic markers: =*mi* and =*tá*. Grzech observes that, unlike prototypical evidential enclitics in related Quechuan varieties, these markers encode not the source of evidence available to the speaker, but rather, information pertaining to the ownership and distribution of knowledge in the discourse. A minimal pair of examples is shown in (7). Note that both sentences contain identical propositional content, but the verb is suffixed by the enclitic =*mi* in (7a) and by =*tá* in (7b). These two markers are also noted by Grzech as having identical semantic, morphosyntactic, and prosodic distributions, but differ in how they relate to so-called “Common Ground management” (Krifka, 2008); that is, the linguistic encoding of information shared among the discourse participants (i.e., the speaker and the addressee).

7.  **Enclitics in Upper Napo Kichwa (Grzech, 2020: 82)**
  - Kan ushanguimi!
    [kan] usha-ngui**=mi**
    2sg can-2**=mi**
    “You can!” (Uttered as a response to the addressee’s prior statement of their being unable to make the traditional Kichwan drink *Chicha*).
  - Kan ushanguirá!
    [kan] usha-ngui**=tá**
    2sg can-2**=tá**
    “You can!” (Uttered to a novice interlocutor who is making *Chicha* for the first time).

The difference between the scenarios in (7) is as follows: in (7a), the speaker desires to assert authority in their reasoning for making the statement of “You can!”, and thus uses *=mi*, whereas in (7b), the speaker wishes to mark the proposition “You can!” as knowledge shared by the addressee, and thus uses =*tá*. Grzech (2020: 82) concludes that the Kichwan =*mi*/=*tá* alternation manages not evidentiality, but rather “epistemic authority” (Pierson, 1994; Zagzebski, 1996, 2012), with *=mi* encoding “the speaker’s authority over knowledge… [and serving] the speaker to indicate their superior epistemic position with respect to the addressee”. On the other hand, =*tá* is used when the speaker wishes to indicate that this authority lies with both them and the addressee; that is, the information is (at least partially) shared between the two participants in the discourse. This state-of-affairs therefore seems to echo the contrast in speaker-addressee knowledge states associated with *that*-mention in the previous example pair in (6). From these observations, we now develop a hypothesis about *that*-mention: the English overt *that* complementizer is used by a speaker as a strategy for encoding epistemic authority over the content of the embedded proposition, and the null form emerges in any other circumstance.

### Why Mark Epistemic Authority?

The notion of *epistemic authority* post-dates Bolinger (1972), going back to Zagzebski (1996). It is characterized as marking a speaker’s judgement or degree of commitment over the knowledge of the proposition that it marks. It is widely noted as being linked to, but independent from, the category of evidentiality (De Haan, 2001; González et al., 2017; Mushin, 2001; Nuyts, 2006). In the Upper Napo Kichwa data, =*mi* and = *tá* manage a speaker-addressee epistemic asymmetry (Grzech, 2020; Sidnell, 2005: whereas *=tá* indicates epistemic equality of speaker and addressee, *=mi* indicates the epistemic authority/superiority of the speaker). It has been observed that claiming epistemic authority is a pragmatically marked strategy (Keen, 1994). Indeed, looking at languages beyond Quechua, we find that this pragmatic markedness corresponds to morphological markedness: in the Australian language Murrinhapatha, epistemic authority is overtly realized by the morphological suffix –*k*, and, in any other instance, the verb has no special marking (Mansfield, 2019). Going back to the pair of examples in (6) concerning a purported event of a robbery, we note that Bolinger (1972) and Dor (2005) judge the speaker to be more likely to omit *that* in the setting where knowledge of the purported event is shared between the speaker (i.e., the police officer) and the addressee (i.e., the gas station owner), by virtue of the visual Common Ground (i.e., the disarray at the scene; 6a). By contrast, *that* is expressed when the visual evidence (i.e., the absence of any sign of a robbery; 6b) instead leads the speaker to assume that the addressee does not share their own knowledge that such a report has been received (i.e., the information is private). In this case, the speaker has epistemic authority, because they hold a perspective on the information that they believe their addressee *not* to share. This hypothesis has potential to account for prior observations in psycholinguistic literature where *that*-mention occurs with a heavy information load (Ferreira & Dell, 2000; Jaeger, 2010): If a speaker has evidence to believe that they themselves hold more knowledge than their addressee, then they should be likely to provide more information (to compensate for the addressee’s relative ignorance) as compared with when the information is mutually known.

Next, we consider the possible function behind encoding epistemic authority in language in the first place. In other words, why mark epistemic authority at all? In answering this question, we first consider a recent analysis of *that* by Staps and Rooryck (2023), who observe that use of *that*—in both demonstrative and complementizer form—occurs when there is shared context (either linguistic or non-linguistic) between the speaker and the addressee. Staps and Rooryck propose that *that* marks “addressee involvement” in what they characterize as the “shared discourse space” (related to Stalnaker’s early notion of Common Ground). In their account, *that* marks the intersection of two discourse spaces, from the vantage point of the speaker: first, the personal discourse space of the speaker and second, the personal discourse space of the addressee. However, we note here that the motivation for linguistically marking the speaker and addressee’s *mutual* knowledge space (i.e., “Common Ground”) might seem mysterious: why might language explicitly mark discourse that is shared? Contrarily, communication is said fundamentally driven by the need to exchange information that is *not* shared, as discussed by Heller and Brown-Schmidt (2023). In motivating their novel “Multiple Perspectives Theory” of mental states in communication, Heller and Brown-Schmidt (2023: 35, emphasis theirs) remark that “what drives communication are the *differences* between conversational partners, and not mutual knowledge”. Our proposal in which *that* marks epistemic authority follows from Heller and Brown-Schmidt’s (2023) “Multiple Perspectives Theory”: We consider complementizer *that* to mark a speaker’s *private* knowledge (*pace* Staps & Rooryck)—thus, a perspective that (they believe) the addressee does not share. However, this marking serves the functional purpose of alerting the addressee to the fact that their own knowledge is about to be updated. In other words, epistemic authority serves as an overt signal to the listener to pay due attention and to prepare for such an update. This maximizes the chance of the communicative exchange being successful.

## EVALUATING THE PROPOSAL

### Forced Choice Task

Our first goal is to substantiate the judgements of Bolinger (1972) and Dor (2005) quantitatively. To do so, we constructed an additional five pairs of scenarios further to the one in (6) and used these items as critical stimuli in a forced choice experiment. In this task, participants were asked to read a context scenario and respond to a question based on the prior context by choosing from a set of four possible options (to be discussed shortly). We manipulated context, with two levels based on Bolinger (1972). In the common ground condition (corresponding to 6a), the speaker and the addressee hold mutual knowledge of the information reported in the embedded clause, as established visually. The speaker therefore has good reason to believe that their perspective of the situation overlaps with the addressee’s. In the speaker authority condition (corresponding to 6b), the oblivion of the addressee leads the speaker to consider the information they hold as being private (i.e., the addressee seems to hold a different perspective of the situation). In turn, the addressee might expect or require information from the speaker as to the purpose of the interaction. An example item is shown in (8).

8.  **Example context pair**
  Mountain Rescue services receive notification of a family trapped on a hillside in stormy weather. They set off to rescue them immediately. {When they arrive, fog has rolled in and visibility is poor, and the family is overjoyed to see Mountain Rescue! common ground/When they arrive, the weather is sunny, and the family is shocked to see Mountain Rescue suddenly arrive! speaker authority}.

After reading the context, participants were asked to respond to the question, of the format “*What is {SPEAKER} most likely to say to {ADDRESSEE}?*”. Of the response options provided, two were related to the context story and contained clausal embedding; importantly, one of these options used an overt complementizer and in the other, no complementizer was present. These were coupled with two other options, which did not involve clausal embedded and were syntactically well-formed but nonsensical based on the prior context story (these were included to detect any participants who were not reading the context carefully). The questions and response options that were coupled with the context in (8) are shown in (9).

9.  **Response to the context in (8)**
  **Question:** What is Mountain Rescue most likely to say the family?
  **Answer:**
  1. We heard that you were trapped here.
  2. We heard you were trapped here.
  3. I wonder how many cars are parked in the parking lot.
  4. I wonder how many cars there are in the parking lot.

#### Materials.

We constructed six context sets following the format in (8). One of them was based on Bolinger’s (1972) original exemplar scenario shown in (6). The other four all comprised different figures of authority (high school principal, town sheriff, fire department, medical consultant) who received a report of an event, which transpired to be accurate (thus, the proposition is known to both speaker and addressee common ground) or inaccurate (thus, accessible to the speaker only; speaker authority). Each context was followed by a question of the format based in (9) and the answer set consisted of a pair that were identical except for presence or absence of an overt complementizer, using matrix verbs of reporting which allowed for both options (*hear*, *gather*, *understand*), plus two nonsensical options. The order of presentation of the total four options was counterbalanced across items. The full set of materials is provided in Appendix A.

The six items were divided between two lists (within-subjects): each participant saw three items in each of the two conditions. These critical items were coupled with eight filler items, each of which comprised a context story followed by the question “*What is {SPEAKER} most likely to say to {ADDRESSEE}?*” with four options, none of which involved clausal embedding. The total of fourteen items were presented in a pseudo-randomized order (no two critical items were adjacent).

#### Method.

The experiment was administered via the web-based platform *IbexFarm* (Drummond, 2013). For each item, the context was presented first, and, after reading it, participants pressed spacebar to advance to the next screen, where the question and four options were presented.

#### Power Analysis.

To determine sample size, we carried out a priori power analysis using the packages *lme4* (Bates et al., 2015) and *simr* (Green & Macleod, 2016), in the *R* programming software (version 4.0.2.). Following Bhatia and Dillon (2022), we estimated effect size by first carrying out a pilot study, using the materials detailed in Materials section. The pilot study had 28 participants. We coded each critical trial as 1 when participants selected the overt complementizer (i.e., *that*) sentence form, and 0 otherwise (all of these were selections of the no complementizer form). A logistic effects regression model fit to our pilot data, with random slopes for context for participants and items, yielded a coefficient for the effect of context (i.e., the change in log-odds of selection of *that* sentence form from common ground to speaker authority conditions) of 1.67. Again, following Bhatia and Dillon (2022), we adopted a more conservative estimate of 1.11 (2/3 of this observed effect size). Our subsequent power analysis based on 100 simulations showed that an experiment with 68 participants would have power of 89% (95% CI: [81.17%, 94.38%]) to detect a change in *that* selection within the parameters of our design.

#### Participants.

68 participants were recruited via Amazon’s Mechanical Turk; all identified as native speakers of English (payment was not contingent on response to this question, so there was no incentive not to answer honestly).

#### Results and Discussion.

One participant was excluded from analysis because they responded with a nonsensical answer option on multiple trials (including fillers). As with the pilot study, we coded responses as 1 when the sentence with *that* was selected, and 0 otherwise (all these trials were selections of the no complementizer form except for one; this trial was removed prior to analysis).

**Figure 1.**
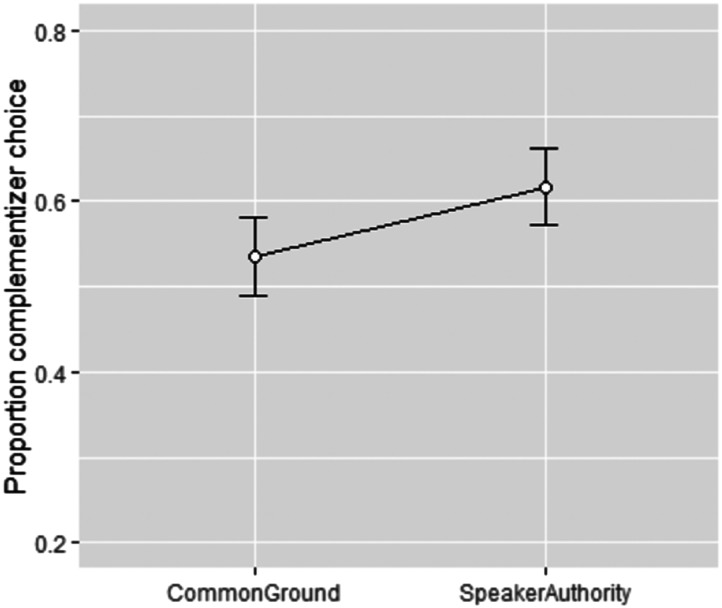
Proportions of complementizer (i.e., overt *that*) sentence selection in the common ground condition as compared with the speaker authority condition. Error bars indicate 95% confidence intervals.

Proportions of complementizer choice are shown in Figure 1. Consistent with the judgements of Bolinger (1972) and Dor (2005), the complementizer sentence form was selected more when the preceding context was speaker authority than when it was common ground (.62 vs. .54; *β* = 0.54; *SE* = 0.26, *z* = 2.05, *p* = .041). This result supports Bolinger’s (1972) judgement in which overt complementizer form is associated with knowledge held exclusively by the speaker. This finding is also consistent with our hypothesis in which *that* signals epistemic authority: a speaker is considered to be more likely to claim this authority when they have a perspective about an embedded proposition their addressee doesn’t seem to share (as in the speaker authority condition), as compared with when both speech act participants are on equal epistemic footing (as in the common ground condition, where speaker and addressee perspectives overlap).

We next test this reasoning further by considering naturalistic production data through corpus analyses.

### Corpus Surveys: Subject Pronoun Form

One factor associated with epistemic authority is subject person form. According to Linguistic literature, epistemic authority is naturally related to the speaker. Marking such authority should go together with an increased likelihood of using first-person pronoun forms (Kittilä, 2019), such as English *I* and *we*. Conversely, it is much less associated with second person pronouns (e.g., *you*), because the speaker is comparatively less likely to claim authority over material which relates to their addressee, rather than themselves. This notion is borne out in Jaminjung and Ngaliwurru, two varieties of a Mirndi language spoken in Australia. Like Upper Napo Kichwa, Jamingjung/Ngaliwurru has an attested system of epistemic marking (Ritz & Schultze-Berndt, 2015). Schultze-Berndt (2017) carried out a survey of a reference corpus, examining how use of the Jamingjung/Ngaliwurru epistemic authority marker =*ngarndi* patterns according to pronoun form of event participants (transitive agents, intransitive subjects, and transitive patients). Consistent with predictions in linguistic literature, Schultze-Berndt found that =*ngarndi* occurred more frequently with first person subject pronouns (34.4% of the tokens) than with second person subject pronouns (7% of the tokens). By contrast, *=ngarndi* occurred most amply with third person pronouns (58.6% of the tokens): this encompasses contexts where the relevant third person is proximate to the speaker in the discourse, or “within the sphere of the speaker” (Dickinson, 2000; Garrett, 2001; Schultze-Berndt, 2017: 212).^1^ Applying this notion to English *that*, one would expect a lower rate of *that*-mention when the embedded clause conveys information about the addressee; specifically, when the subject of the embedded clause is “*you*”, such as in the corpus token example in (10a). Here, the speaker has due reason to assume that the addressee shares the knowledge of their own state of being very happy. In contrast, we expect a higher rate of *that*-mention when the embedded clause contains information about the speaker, signalled via a first-person embedded subject (“*I*”/“*we*”), as in (10b), which the addressee is less likely to share.

10.  **Corpus tokens.**
  - *I seem to recall you were very happy.*      [COCA, Movie, 2012]
  - *I wanted to let you know **that** I am leaving.*   [COCA, Movie, 2018]
                        (Davies, 2008)

Outside of literature of epistemic authority, Ferreira and Dell (2000) have examined how first and second person pronoun forms (“*I*” vs. “*You*”) condition English *that*-production, looking for effects of ambiguity: “*I*” is unambiguously a (nominative-cased) embedded subject, whereas “*you*” could be either an embedded subject or a direct object (if “*that*” is dropped). They used an experimental paradigm where participants were first provided with a prompt and then asked to recall a sentence with a complement clause by speaking it aloud (or to provide completions of sentences). Adopting this type of paradigm to test for epistemic authority, however, faces an inherent challenge: Whereas sentences produced by participants in controlled production settings are necessarily provided to them (by the experimenter/lab), epistemic authority concerns a speaker’s *own* thoughts/beliefs/desires/knowledge. Critically, this means that participants’ communicative goal in this type of controlled setting is to relay information already belonging to somebody else (i.e., the experimenter/the lab/stimuli creator), rather than to report on their own original narrative. Indeed, Ferreira and Dell did not find a consistently reliable effect of *I* vs. *You* on complementizer form. In two subsequent production studies using the matrix verbs *think* and *believe*, Ferreira and Hudson (2011) examined a factor which contributes to epistemic authority—namely, ownership of emotion. They manipulated for each experimental trial whether ownership lay with the speaker or with the addressee, and found a small effect of embedded subject form (*I* vs. *You*), with *that* mentioned more for *I* than for *You* (i.e., patterning in the same direction as the Jamingjung/Ngaliwurru marker *=ngarndi*). At the same time, however, we note that manipulating ownership of emotion enabled only a partial control for epistemic authority, not a complete one. This is because information about the hypothetical behavioral event in question was read aloud for every trial, and therefore always comprised *mutual* knowledge of the speaker and addressee (i.e., Common Ground), rather than private speaker knowledge. We proceed by examining naturalistic production data (cf. Schultze-Berndt, 2017), by conducting a corpus survey to examine the distribution of *that*-mention in embedded clauses with first person and second person subjects. The corpus surveys reported in this paper were each carried out using *Corpus of Contemporary American English* (*COCA*; Davies, 2008). *COCA* is a widely-used database which comprises approximately one billion words of text, from the years 1990 to present, from eight different genres: academic texts, blogs, fiction, magazines, newspapers, transcripts of spoken dialogue, television and movie subtitles, and webpages. We consider specifically embedded clauses because it is these environments alone where the relevant pronouns (i.e., those in the embedded proposition) fall *beneath* the linguistic scope of the hypothesized epistemic authority marker (i.e., *that*), mirroring the state-of-affairs in Jamingjung/Ngaliwurru, where epistemic markers scope over the entire sentence (Schultze-Berndt, 2017). Our searches focus upon two classes of matrix verbs discussed in Dor (2005); both embed complement clauses and permit either null or overt (*that*) complementizer form. The first type is labelled by Dor as “speech act predicates”, and the second type is predicates of “belief, knowledge, and conjecture”. Collectively, these comprise “*that*-optional” verbs (i.e., Dor’s “Type I”; we return to *that*-preferred and *that*-required cases (Dor’s Types II and III) in forthcoming sections. Each survey considers two variables: first, the form of the complementizer (null vs. *that*) used to introduce the embedded clause (identified in the survey via the presence of an embedded subject plus an embedded verb), and second, the person of the embedded subject (first person vs. second person). A complete list of counts for each verb can be found in Appendix B.

#### Survey A: Speech Act Predicates.

A total of 27 verbs of this type are identified by Dor (2005: 351–2) and defined as “denoting assertive illocutionary acts”: *say*, *tell*, *claim*, *assert*, *affirm*, *state*, *argue*, *inform*, *remind*, *predict*, *suggest*, *disclose*, *reveal*, *indicate*, *intimate*, *insist*, *hypothesize*, *admit*, *confess*, *divulge*, *show*, *demonstrate*, *make clear*, *point out*, *contend*, *remark*, and *note*. We searched for all instances of these verb lemmas in *COCA* and identified a total of 37,393 token sentences where the embedded subject was either first person (singular *I* or plural *we*)^2^ or second person (*you*; English second person is underspecified for number). Each of these were tagged as either containing an overt complementizer (“*that*”) or no complementizer (“null”). Of the 21, 201 sentence tokens with embedded first-person subject pronouns, the embedded clause was introduced by an overt complementizer in 6083, or 28.7% of them. This proportion, however, significantly decreased for embedded second-person pronouns: of the 16,205 tokens of this type, the embedded clause had an overt complementizer in 3400, or 21% of them (*χ*^2^ = 285; *p* < .0001). We summarize this output in Table 1.

**Table 1.** Summary of token counts of speech act predicates with first- and second-person embedded subjects.

		*Ø*	*that*	% *that*
Embedded subject form	**1^st^ person (*I*/*we*)**	15,118	6083	**28.7%**
	**2^nd^ person (*you*)**	12,804	3401	**21%**

This drop in overt complementizer usage with second-person subjects by comparison with first-person subjects is analogous to the decrease in the use of the epistemic authority marker in Jamingjung/Ngaliwurru found by Schultze-Berndt (2017) and supports our hypothesis in which English *that* signals epistemic authority of the speaker. Of the 27 verbs we surveyed, this pattern in which *that* is more prevalent with first- than with second-person pronouns held for 17 of them. Seven showed the reverse pattern, qualitatively (*affirm*, *remind NOUN*, *reveal*, *insist*, *confess*, *demonstrate*, and *point out*). A further three verbs yielded no tokens with either first- or second- person embedded subject pronouns whatsoever (*hypothesize* and *divulge*), or no tokens with null complementizer form (*remark*).

#### Survey B: Predicates of Belief, Knowledge, and Conjecture.

We turn next to the second matrix verb type identified by Dor (2005); namely, predicates of belief, knowledge, and conjecture. These are defined by Dor (p. 352) as “entail[ing] a commitment on the part of their cognitive subjects to the truth of the proposition denoted by the embedded clauses”. Dor lists 22 verbs of this category: *believe*, *think*, *conclude*, *suppose*, *agree*, *maintain*, *fancy*, *presume*, *assume*, *know*, *be aware*, *recall*, *remember*, *forget*, *find out*, *discover*, *notice*, *realize*, *guess*, *conjecture*, *imagine*, and *figure*. As with Survey A, we searched for all instances of these verbs in *COCA*, finding 86,566 tokens with embedded first- or second-person subject pronouns. These were tagged according to the same criteria as Survey A. Here, there were a total of 54,639 tokens with embedded first-person subject pronouns. The embedded clause was introduced by an overt complementizer in 5342, or 9.8% of them. As we found for the speech act predicates in Survey A, this proportion was reduced for embedded second-person pronouns: of the 31,927 tokens of this type, the embedded clause had an overt complementizer in 2566, or 8% of them, a small but significant decrease (*χ*^2^ = 73.3; *p* < .0001); we summarize this in Table 2. All 22 of the verbs we surveyed followed the overall patten. Therefore, we find once again that English *that* patterns akin to epistemic authority markers in Jamingjung/Ngaliwurru.

**Table 2.** Summary of token counts of predicates of belief, knowledge, and conjecture with first- and second-person embedded subjects.

		*Ø*	*that*	% *that*
Embedded subject form	**1^st^ person (*I*/*we*)**	49,397	5342	**9.8%**
	**2^nd^ person (*you*)**	29,361	2566	**8%**

#### Other Effects: Factivity and Matrix Subjecthood.

Lastly, we discuss two additional factors which have been discussed in connection to *that*-optionality. First, one might consider the role of the matrix subject form (e.g., *I*/*we* vs. *You*). Previous studies have observed that lower rates of *that*-usage under non-factive matrix verbs such as *think* and *guess* for first person matrix subjects than for any other types of NP subjects (Thompson & Mulac, 1991; Torres Cacoullos & Walker, 2009). We note however, that an account based on epistemic authority does not make any specific predictions regarding effects of the matrix subject, because these elements are not a part of the (embedded) proposition which is/isn’t marked by *that*. Conversely, they fall outside of the scope of the hypothesized epistemic authority marker (i.e., *that*), although they may be associated with authoritative meaning in other ways (e.g., deontic authority)^3^.

Second, recent experimental work has established that matrix factive verbs, namely, verbs which presuppose the truth of their embedded complements (cf. Kiparsky & Kiparsky, 1970; et seq.), are judged by speakers of English to be less acceptable with *that*-omission as compared non-factive verbs (Bîlbîie et al., 2023). In short, *that*-optionality is sensitive to factivity. To assess whether the observed interaction of *that*-mention frequency with embedded 1^st^ vs. 2^nd^ person subjecthood might be an effect also related to factivity, we examined factive and non-factive verbs side-by-side. We looked at the subset of the verbs from our Surveys A and B which are listed as either factive or semi-factive in Hooper (1975) and Hooper and Thompson (1973) and compared these with those which are listed by these authors listed as non-factive. Consistent with Bîlbîie et al. (2023) we found that the non-factive verbs (*think*, *say*, *believe*, *suggest*, *guess*, *suppose*, *insist*, *admit*, *figure*, *state*, *claim*, *imagine*, *agree*, *indicate*, *point out*, *argue*, *presume*, *demonstrate*, *assert*, *maintain*, *affirm*, *remark*, *contend*, *intimate*, *hypothesize*, *predict*, *divulge*) yielded a lower rate of *that*-mention (11.7%) compared with the subset characterized as factive or semi-factive (*know*, *remember*, *realize*, *notice*, *find out*, *discover*, *note*, *reveal*; 29.5%). This is line with our earlier discussion of non-homogeneity of *that*-optionality across different kinds of embedding verbs (Introduction). Importantly, we also find that effects of 1^st^ vs. 2^nd^ person subjecthood regarding *that*-mention hold for both the non-factive and the factive/semi-factive groups. For non-factive verbs, 1^st^ person embedded subjects were preceded by overt *that* 12.7% of the time, versus 10.6% of the time for 2^nd^ person subjects (*χ*^2^ = 114.8; *p* < .0001). For factive verbs show the same pattern, with 1^st^ person embedded subjects preceded by overt *that* 30.7% of the time, versus 28.3% of the time for 2^nd^ person subjects (*χ*^2^ = 5.2; *p* = .021). This suggests that the effect of 1^st^/2^nd^ person subjecthood holds independently of verb factivity. However, we also observe an interaction where the effect of person upon that-mention is smaller for factive/semi-factive compared with non-factive verbs (*χ*^2^ = 55.6; *p* < .0001), a matter which we leave open.

### Summary and Further Discussion

To summarize, we find that the overt complementizer *that* is more frequent with a first-person embedded subject, compared with second person. This holds for both types of verbs in Dor (2005). The decrease in complementizer usage for second person subjects cannot be directly accounted for under psycholinguistic theories of *that*-mention which appeal to information load, such as Uniform Information Density (Jaeger, 2010). We note that this comparison allows us to control for information load (cf. Jaeger, 2010) as best possible within the parameters of our search criteria: first and second person pronouns have been argued to encode analogous levels of morphosyntactic information (Béjar & Rezac, 2009; Bonet, 1991) and are equally discourse central (Warren & Gibson, 2002). By comparison, third person pronouns are slightly less morphosyntactically complex and slightly more discourse peripheral, meaning that they cannot be compared with first/second pronouns while simultaneously controlling information density.

Our results are, however, predicted under the epistemic authority hypothesis: Epistemic authority most naturally relates to the speaker, and therefore, to use of first-person pronouns (Kittilä, 2019) more so than second person pronouns, because the speaker is comparatively less likely to claim authority over the relevant proposition when the addressee (i.e., “*you*”) is referenced. This effect indeed appears to be characteristic of optional *that*-mention as opposed to being a more general artefact of overt versus non-overt embedding, which extends beyond *that*^4^. For instance, we find no evidence of an analogous person effect for the distribution of (embedded) object relative clauses as a function of whether a *wh* filler is overt (e.g., *The dog [**which** the cat is following__]*) or null (e.g., *The dog [the cat is following__]*): Of a total of 79,589 instances in *COCA* of object relative clauses with 1^st^ or 2^nd^ person subjects, only 931 of them (1.2%) had an overt *wh* filler, with no significant effect of person (*χ*^2^ = .14; *p* = .71).

Before turning to how epistemic authority relates to other factors connected to *that*-mention (Connections With Newsworthiness and Information Load: Corpus Survey C and General Discussion sections), we briefly review our proposal. We contend that one of the factors conditioning *that*-mention, and potentially the core interpretative factor, is the need or desire of the speaker to signal a superior state of knowledge over the clausal proposition embedded beneath the complementizer. We hypothesize that a key factor guiding a speaker’s choice between uttering an embedded clause with an overt complementizer such as (11a), versus one without such as (11b), is the extent to which they perceive themselves as knowing more about the embedded proposition (e.g., “the cat is following the dog”), compared to their addressee.

11.  English complementizer omission [= (2)]
  - *I think [**that** the cat is following the dog].*   OVERT COMPLEMENTIZER
  - *I think [the cat is following the dog].*    NO/NULL COMPLEMENTIZER

Under this proposal, one of the uses of *that* is to encode a superior knowledge state of the speaker vis-à-vis the addressee—thereby, a perceived difference (according to the speaker) in perspectives of the conversational participants. We hypothesize that choice of using *that* would include both linguistic factors, such as whether information contained in the embedded clause is old or new to the discourse, as well as extra-linguistic factors, such as personality traits, interpersonal dynamics, and the nature of the social setting where the conversation takes place (Gipper, 2015; Grzech, 2020). In the next section, we revisit the first of these: the discourse status of the information contained in the embedded clause.

## CONNECTIONS WITH NEWSWORTHINESS AND INFORMATION LOAD: CORPUS SURVEY C

In this section, we consider connections between interpretative context of that, and psycholinguistic accounts of *that*-mention, such as Jaeger’s (2010) theory of *Uniform Information Density*. These accounts draw upon widely replicated observations that embedded clauses marked by overt *that* contain higher information loads as compared those with the null complementizer form (see discussion in Psycholinguistic Accounts of *That*-Optionality section). Jaeger (2010) shows that embedded clauses with longer utterance form are more likely to be marked by overt *that* than those with shorter utterance form. More simply put, the more words the embedded clause contains, the greater the probability that *that* is uttered: this marks a strategy used to distribute denser information signal across more lexical items (Jaeger, 2010; Levy & Jaeger, 2007). Importantly, as noted by Jaeger (2010: 2), the use of more words, or a more complex utterance form, is affected by how new (or old) to the discourse the information in the utterance is (Arnold et al., 2000): newer information is associated with longer form. From this platform, we hypothesize that there exists a two-way association of informational newness/length with epistemic authority, as follows: first, it is precisely with newer information that a speaker is most likely to mark their epistemic authority, since newer information is (almost by definition) information that the addressee has less prior knowledge of compared with the speaker. Second, the encoding of epistemic authority would naturally be coupled with longer utterance form, as the speaker needs to supply more information to compensate for their addressee’s relative lack of knowledge (by comparison with the speaker).

We put this reasoning to the test by carrying out a third corpus survey, focusing this time on the use of a discourse marker which signals the presence of new information. Fortunately, such a marker has previously been identified as serving this purpose: *actually*, as used in environments such as the exchange in (12). Here, speaker A expresses a statement from which speaker B marks a deviation. Lenk (1998: 160–1) contends that *actually* in such circumstances “expresses that the following [material] will be slightly (moderately/definitively) different from the expected normal course of the conversation” and “marks the opinion expressed as not given before”.

12.  **Example use of *actually***
  - It is raining.
  - Actually, it is snowing.
              (Smith & Jucker, 2000: 222)

Building on Lenk (1998) and Smith and Jucker (2000: 212) analyse *actually* as “signaling deviation […] from the attitude on the floor”, along the dimension of newsworthiness (as well as speaker commitment and evaluation): according to Smith and Jucker, use of *actually* marks the speaker’s estimation of the newsworthiness of a proposition. In view of our proposal of complementizer *that* as a marker of epistemic authority, we therefore predict that complement clauses marked with *actually* will show greater use of the overt *that* complementizer form than those without.

As noted by Lenk (1998) and Smith and Jucker (2000), *actually* is commonly used in close proximity to the phrase “*I think*”. With this in mind, we focused our search on instances of the verb *think* with a first-person matrix subject (i.e., *I* or *we*, following our studies A and B) which are followed by complement clauses. Of interest is the comparison between “*I*/*we think*” and “*I*/*we actually think*”: as discussed, we predict a higher rate of that-mention following the latter, where use of *actually* signals upcoming new information. There is, however, a viable alternative state of affairs: it is possible that the presence of *any* discourse marker (or adverbial material of any kind) preceding a matrix verb like *think* could alter rates of complementizer mention, for a reason entirely independent of the pragmatic contribution of the discourse marker itself. Therefore, to isolate the pragmatic function of *actually* as marking *new* information, we also looked for first-person instances of *think* preceded by a similar discourse marker, but one which does *not* serve to mark information as new: *totally* (i.e., “*I*/*we totally think*”). Instead of dedicatedly marking upcoming information as new, *totally* signals that that the information ought to be shared between all conversation participants: Beltrama (2018: 16) analyses *totally* as a “common-ground managing operator” paraphrasable as “*unquestionably*”, which marks the speaker’s belief that the embedded proposition should be “a part of the speaker-addressee common ground”.

**Figure 2.**
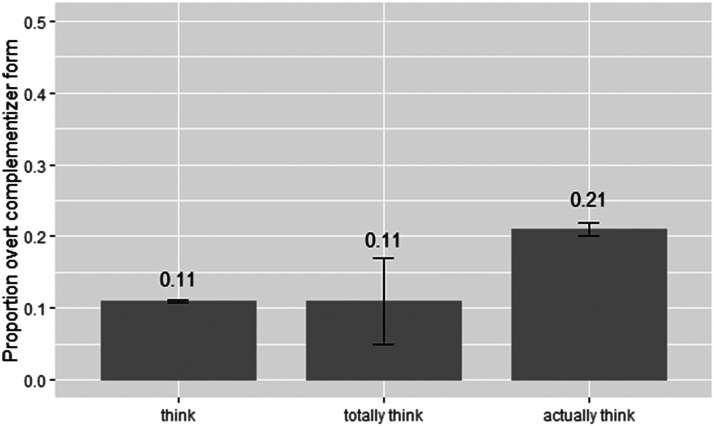
Proportion of tokens of “*think*” (*n* = 884, 222), “*totally think*” (*n* = 75), and “*actually think*” (*n* = 2301) with first-person matrix subjects and followed by an embedded complement clause, for which the embedded clause is marked with *that*. Error bars show estimated ±1 *SD* following Cowan (1998).

We searched *COCA* for all (both past and present tense) instances of (i) “*think*” plain (ii) “*totally think*”, and (iii) “*actually think*” with a first-person subject which were followed by complement clauses (i.e., an embedded subject plus an embedded verb). The instances of “*think*” plain totalled 884,222 (of which in 96,979, the complement clause was marked with overt *that*). There were 75 instances of “totally think” (8 with overt *that*), and 2031 of “actually think” (422 with overt *that*); these results are shown in Figure 2. Before considering instances of *actually*, the first goal of our analysis was to ascertain whether and to what extent *totally* alters proportion of *that*-mention by comparison with plain *think*. Importantly, we find no evidence that it does: the proportions for “*think*” and “*totally think*” were akin (11%; *χ*^2^ < .0001). This is consistent with a view that *totally*, while nonetheless contributing pragmatic information (Beltrama, 2018), does not alter rate of *that*-mention in comparison with *think* alone (i.e., doesn’t either decrease or increase it). We therefore turn to comparing “*totally think*” with “*actually think*”: here, we find that rate of *that*-mention almost doubles when *think* is preceded by *actually* by comparison with totally (*χ*^2^ = 3.95; *p* = .047), supporting our hypothesis that the presence of new information as marked by *actually*—elevates rate of overt complementizer form.

For completeness, we also considered occurrences of clausal embedding under *actually* + *think* with other types of matrix subjects. Here, we found a total of out of 3,809 tokens of which 756, or 24.8%, included overt *that*. Again, this was a significant increase by comparison with *totally* + *think*, which yielded 105 tokens, of which 11 (10.4%) had overt *that* (*χ*^2^ = 5.12; *p* = .023).

Collectively, these results support a view where complementizer *that* is more frequently used when information in the embedded proposition is also marked as new to the discourse. This connects our proposal in which *that* marks a speaker’s epistemic authority with the results of Jaeger (2010), showing that mention of *that* is elevated as new information load increases, while simultaneously encompassing interpretative content. Next, we return to discussing types of constructions where complementizer *that* cannot be omitted, motivating how the absence of *that*-optionality in these environments follows from our epistemic authority account.

## GENERAL DISCUSSION

We propose that the core interpretative component of complementizer *that* is as a marker of epistemic authority (Zagzebski, 1996; a.o.), which signals that the speaker assumes they know more (or projects themselves as knowing more) about the content of the embedded proposition as compared to their addressee. Functionally, this serves a communicative goal of signalling information privileged to the speaker (cf. Heller & Brown-Schmidt, 2023), in order to alert the addressee to expect to update their own (comparatively inferior) knowledge space.

This account extends to explaining a long-discussed observation in syntactic literature: *that* can only *ever* be omitted when the embedded clause is in canonical object position (i.e., when it surfaces as a complement, and appears to the right of matrix verb). For clauses in subject position like in (13a) or fronted sentence-initial position as in (13b), *that*-mention is grammatically required (Davies & Dubinsky, 2009; Delahunty, 1983; Rizzi, 2000; Rosenbaum, 1967; Stowell, 1981, a.o.).

13.  **No *that*-omission in sentence-initial or fragment clauses.**
  - Subject clause
     [*(That) the cat is following the dog] is well-known.
  - Sentence-initial clause
     [*(That) the cat is following the dog], I already realized.

It is well established in linguistic literature that the sentence initial or grammatical subject position of a sentence is reserved for information that continues discourse “topic” of an utterance (Chafe, 1994; Kuno, 1976; Lambrecht, 1994), referring to information that is backgrounded, or which is directly inferable from the context of the utterance (Miller, 2001). The information appearing in this position is accessible to both participants in the conversation (i.e., speaker and addressee). In contrast, the information that follows this is packaged grammatically as discourse-new information, in the sense that it occurs in syntactic object position. In the terminology of Krifka (1992), this comprises the “comment” about the initial subject/topic, which provides updated information about it. Information in sentence initial position is assumed to be known to both the speaker and the addressee - whereas information in comment (post-initial) position tends to be information which updates the mutual knowledge between speaker and addressee. In short, the speaker and addressee *both* have knowledge of the subject/topic. However, the speaker necessarily has *more* knowledge about it than their addressee (i.e., holds epistemic authority) and believes the addressee not to have the same perspective on it as they themselves do—hence, the speaker’s very act of updating the addressee’s knowledge of it by providing a new comment. Thus, when this initial information is linguistically packaged in the form of an embedded, a speaker’s superior knowledge state is de facto marked.

A second instance of obligatory *that*-mention arises in the form of question response fragments^5^: *that*-omission is ill-formed in clausal fragment answer to a *wh* question like in (14) as shown experimentally by Merchant et al. (2013).

14.  **No *that*-omission in clausal fragment answers.**
  Q.  *What did you already realize?*
  A.  *(That) the cat was following the dog.

The obligatoriness of *that* in this instance follows from our account above: The conversational move of asking a question signals some lack of knowledge. In turn, answering a question serves the conversational goal of filling a gap in the knowledge state of one’s conversational participant. In terms of the information that gets encoded linguistically (i.e., via the very syntax/pragmatics of a question plus response pair), the speaker/question-answerer knows something more than their addressee/question-asker—hence the original conversational move by the latter.

Next, we consider the kinds of matrix embedding verbs for which *that*-mention is generally preferred (and for some speakers, required) over *that*-omission. These comprise the “that-preferred” and “that-required” predicate categories (i.e., Dor’s (2005) “Type II” and “Type III”, respectively). They include manner-of-speaking predicates (e.g., *whisper*, *murmur*), predicates of instrument communication (e.g., email, fax), emotive predicates (e.g., *be proud*, *be amazed*) and performative verbs (e.g., *declare*, *pronounce*). Many of these verbs are so-called “factive” predicates (Kiparsky & Kiparsky, 1970); namely, predicates for which the truth of their clausal complements is presupposed when the sentence is uttered. For example, the verb *think* is non-factive, because it does not introduce any presupposition regrading the truth of the proposition it selects for. On the other hand, verbs like *regret* are factive, because they presuppose that the proposition in the embedded clause is true. Recent experimental work has found that embedded clause complements of factive verbs are judged by speakers of English to be less acceptable with *that*-omission as compared non-factive verbs (Bîlbîie et al., 2023), indicating that factivity is a key factor in conditioning acceptability of *that*-omission.

We reason that this connection may be due at least in part to connections between factivity and the linguistic category of evidentiality (Dahlman & van de Weijer, 2022; Simons, 2007). Importantly, Simons (2007) argued that matrix factive verbs which take clausal complements (introduced by *that*) fulfil an evidential function, where the clausal complement itself holds “main point” (of utterance) status, and the verb introducing this complement is a type of lexical evidential marker (see also Asudeh et al., 2017). As we discussed in Semantic-Pragmatic Accounts of *That*-Mention section, evidentiality is strongly linked with epistemic authority, as follows: If a speaker has strong evidence for uttering a sentence, then it is likely that they simultaneously assume authority for it (although not categorically necessary). Schultze-Berndt (2017: 190) remarks that “very frequently, and rather unsurprisingly, the speaker’s epistemic authority is indeed based on […] evidence for the reported event”. We therefore suggest that “factive” verbs behave akin to lexical evidentials, insofar as they indicate a speaker’s evidence for, or stance towards, their complement. Thus, the marking of epistemic authority (i.e., *that*-mention) is strongly preferred for many speakers of English in this context.

Finally, we consider other factors in communication which can influence a speaker’s decision to either utter or omit *that*. One widely discussed feature of *that*-mention is register: Elsness (1984) identifies *that*-mention as being more frequent in formal speech, and this has been connected to the need/desire of a speaker to play a more knowledgeable and serious persona (Eckert, 2018). A second widely discussed observation surrounding *that*-mention is its potential to alleviate local parsing ambiguities (Bolinger, 1972; Rohdenburg, 1996; a.o.) and thus increase chances of successful communication (Grice, 1975). For example, verbs such as “accept” in (15) that can take either a nominal complement, like “the prize” in (15a) or a clausal complement, as in (15b).

15.  **Ambiguity in postverbal NP role**
  - *The man accepted [the prize]_NP_.*            = Direct object
  - *The man accepted [**(that)** the prize was not going to him]_CP_.* = Embedded subject
                         (Trueswell & Kim, 1998: 103)

In a sentence like (15b), in which “the prize” is semantically compatible either as a direct object of “accept” (cf. 15a) or as a sentential subject, *that*-mention can serve to mitigate a garden-path effect for the addressee/listener: It signals the onset of clausal complement, therefore alleviating a temporary mis-parsing of “the prize” as a direct object. We hypothesize that epistemic authority is an interpretative consequence of *that*-mention even where not the primary factor in guiding a speaker’s decision of whether to utter it in the first place. In other words, clausal constructions with *that*-mention give rise to a meaning in which epistemic authority is present. Notably, we would expect *that*-mention to be dis-preferred in environments where epistemic authority is afforded to the *addressee* rather than the speaker: namely, *wh* questions (cf. Heller & Brown-Schmidt, 2023). This is because marking a proposition privileged knowledge of the addressee and of the speaker at the same time would create a meaning where the speaker both appears as both *knowledgeable* and *ignorant* as to its contents—a situation which goes against common logic. This situation plays out in certain types of embedded *wh* dependency contexts, yielding a constraint known in linguistic literature as a “complementizer-trace” or “that-trace” effect (Culicover, 1993; Perlmutter, 1968). For example, in an English embedded subject *wh* question like in (16), *that* must be dropped as in (16a), and cannot be overt, as in (16b).

16.  **Complementizers and subject dependency formation**
  - Null complementizer
    *Who did he say [ ___ hid the rutabaga]?*
  - *That*-mention
    *Who did he say [**that** ___ hid the rutabaga]?
                        (Perlmutter, 1968: 214–5)

This ill-formedness of extracting a subject out of a *that*-clause ties in with a view in which *that* contributes a meaning pertaining to epistemic authority. The *that*-trace effect primarily concerns clause-initial subject extraction (i.e., extraction of backgrounded information). It impacts extraction of objects (i.e., focal information) only to a small (but robust) extent (Sobin, 2002; Tollan & Palaz, 2021): Constructions like “*What did he say **that** they hid?*” are rated lower than “*What did he say they hid?*”, relative to a non-extraction baseline. We leave a full analysis of these observations and potential further connections with speaker/addressee knowledge states to future research.

## CONCLUSION

We have motivated a new proposal in which complementizer *that* encodes epistemic authority on the part of the speaker and tends to be used when a speaker wishes or needs to mark that they have more knowledge than their addressee of the information in the embedded complement. In developing this theory, we bring together observations in previous research which have established that (i) *that* is more commonly mentioned in circumstances where the speaker utters a clausal complement containing a high information load (Ferreira & Dell, 2000; Jaeger, 2010; Rohdenburg, 1996; Roland et al., 2006), and also (ii) *that* carries meaning (Bolinger, 1972; Dor, 2005; Hiroe, 1999; Rooryck, 2001; Thompson & Mulac, 1991; Yaguchi, 2001). We reason that mention of *that* is guided by whether a speaker considers themselves to have (or want to project themselves as having) more knowledge of information packaged by the upcoming clause, compared with their addressee: namely, it marks *epistemic authority* (Grzech, 2016, 2020; Keen, 1994; Kittilä, 2019; Mansfield, 2019; Pierson, 1994; Schultze-Berndt, 2017; Sidnell, 2005; Zagzebski, 1996, 2012). This is associated with constructions where the speaker is talking about themselves (via use of a first-person pronoun) as opposed to their addressee (via a second-person pronoun), and environments where new or contrary information is being introduced to a dialogue. Finally, some linguistic environments do *not* grant the speaker the same choice in uttering or omitting *that*: In particular, embedded clauses in subject or sentence-initial position require *that*-mention, as do fragment responses to *wh* questions. These environments are ones in which the speaker’s utterance responds to a linguistically encoded absence in knowledge of their addressee (via inherent information structure of a sentence, or via a prior conversational move), and therefore disallow indeterminacy vis-à-vis epistemic authority. Broadly, this inquiry highlights how understanding factors which guide a linguistic optionality can be informed by consideration of linguistic environments where such a decision is unavailable, and vice versa.

## ACKNOWLEDGEMENTS

We thank Robin Andreasen and Daphna Heller for insightful discussions about this project. This research was supported by funding from the University of Delaware.

## FUNDING INFORMATION

This research was supported by funding from the University of Delaware.

## AUTHOR CONTRIBUTIONS

**RT**: Conceptualization; Formal analysis; Investigation; Methodology; Writing – original draft; Writing – review and editing. **BP**: Investigation; Writing – review and editing.

## DATA AVAILABILITY STATEMENT

The data that support the findings of this study are available from the first author upon request.

## Notes

^1^ English *that* has likewise been observed to occur more frequently with 3^rd^ person subject pronouns (i.e., *he*/*she*/*they*) than with 1^st^ person pronouns (Elsness, 1984; Ferreira & Dell, 2000; Jaeger, 2010; Roland et al., 2006).^2^ By comparing second person number-neutral pronoun *you* with both singular *I* and plural *we* simultaneously, we control for plurality within the parameters of our searches. Plural number is analysed as being more semantically complex than singular number (Bennett, 1974; Chierchia, 1998; Schwarzschild, 1996) and as such, the typically adopted *I* vs. *you* contrast is not a linguistically minimal one.^3^ To assess whether effects of embedded subject person might be reduced to effects of matrix subject person, we searched independently for instances of the verbs in survey lists A and B with first- and second-person matrix subjects, as well as with first- and second-person embedded subjects. We found 32159 instances of 1^st^ person + 1^st^ person (8% with *that*), 23277 instances of 1^st^ person + 2^nd^ person (7.1% with *that*), 7697 instances of 2^nd^ person + 1^st^ person (10.7% with *that*), and 7642 instances of 2^nd^ person + 2^nd^ person (10.3% with *that*); thus, fewer instances of *that*-mention for 2^nd^ person embedded subjects than for first person embedded subject for both matrix subject forms.^4^ Previous literature in linguistics has drawn semantic and syntactic parallels between complementizer *that* and other functions of *that*: namely, as a demonstrative determiner (Staps & Rooryck, 2023) and as a relativizer (Douglas, 2017). The matter of whether these two further uses of *that* might also mark epistemic authority remains open.^5^ A further instance of obligatory that-mention which has received attention in linguistic literature is the case of exclamatives such as “*That he would leave!*”. As motivated by Beijer (2002), exlamatives function as a means of expressing a speaker’s emotional attitude towards a proposition via intonation rather than via propositional form (as in “*I am very surprised that he would leave!*”; Beijer, 2002: 11). Thus, the speaker introduces information previously unknown to their addressee; namely, their attitude. As with the case of fragment answers, the speaker marks superior knowledge (of their attitude) as compared with their addressee via *that*.

## APPENDIX A: LIST OF CRITICAL STIMULI FOR THE FORCED-CHOICE TASK

1. Police Officer Bryant receives a phone call about a robbery at a local gas station and sets off immediately. When he arrives at the gas station, he sees {that the whole place is in disarray, and finds the owner injured and distressed. _common ground_/that the whole place is neat and tidy, and the owner is surprised to see a police officer. _speaker authority_}
  **What is Officer Bryant most likely to say to the owner?**
  *I heard that you’ve been robbed.*
  *I heard you’ve been robbed.*
  *I just saw a tsunami outside.*
  *I just heard a tsunami outside.*
2. Mountain rescue services receive notification of a family trapped on a hillside in stormy weather. They set off to rescue them immediately. {When they arrive, fog has rolled in and visibility is poor, but the family is overjoyed to see mountain rescue! _common ground_ /When they arrive, the weather is sunny, and the family is shocked to see mountain rescue suddenly arrive! _speaker authority_}
  **What is Mountain rescue most likely to say the family?**
  *We heard that you were trapped here.*
  *We heard you were trapped here.*
  *I wonder how many cars are parked in the parking lot.*
  *I wonder how many cars there are in the parking lot.*
3. Matt is a high school principal. One day, he receives a report that a student has been upset in a math class and calls for the student to go to his office. When the student arrives {she is crying, and relieved to have somebody to talk to. _common ground_ /she is not at all upset and doesn’t seem to understand why she has been called to the office. _speaker authority_}
  **What is Matt most likely to say to the student?**
  *I understand you’re upset.*
  *I understand that you’re upset.*
  *I made a sandwich for lunch.*
  *I made a sandwich to eat at lunch.*
4. Carla is a medical consultant, who runs her own practice. She discovers that one of her patients has broken a leg and goes to visit them. When Carla arrives at the patient’s home, {the patient is struggling to walk, and is relived to see Carla. _common ground_ /the patient seems absolutely fine and doesn’t understand why Carla is there. _speaker authority_}
  **What is Carla most likely to say to the patient?**
  *Do you want to go to South Africa?*
  *Do you want to travel to South Africa?*
  *I gather that you broke your leg?*
  *I gather you broke your leg?*
5. The town warden hears a report of vandalism at a local park. He sets off for the park, and finds {garbage everywhere, and angry-looking locals. _common ground_ / no signs vandalism, and the locals surprised to see the town warden. _speaker authority_}
  **What is the town warden most likely to say to the locals?**
  *I do not like trees.*
  *I do not appreciate trees.*
  *I heard the place has been vandalized.*
  *I heard that the place has been vandalized.*
6. The fire department is called to a burning shed in a local garden center. When they arrive at the scene, {the shed is completely burnt to the ground, and the staff member is relieved to see them. _common ground_ /the shed is intact, there are no signs of any damage, and the staff member is surprised to see them. _speaker authority_} The chief fire officer turns to the staff member.
  **What is the office most likely to say to the staff member?**
  *I just got back from the grocery store.*
  *I just got back from the local shop.*
  *I heard that a fire started here earlier today.*
  *I head a fire started here earlier today.*

## APPENDIX B: TABLES WITH VERB LEMMAS AND COUNTS FOR CORPUS SURVEYS A AND B

**Table B1.** Survey A: Speech act predicates.

**Verb lemma**	**1st person subject**		**2nd person subject**		**Total**
	**Ø**	***that***	**Ø**	***that***	
*say*	11412	3192	8534	1506	24644
*suggest*	1397	788	2579	757	5521
*show*	470	224	660	272	1626
*insist*	503	420	181	174	1278
*admit*	444	330	206	150	1130
*state*	274	205	229	122	830
*claim*	174	161	194	106	635
*tell NOUN*	195	102	132	45	474
*confess*	136	108	6	17	267
*indicate*	17	86	34	100	237
*point out*	31	129	11	48	219
*argue*	25	126	14	31	196
*demonstrate*	6	29	5	32	72
*note*	4	46	6	11	67
*reveal*	16	19	1	14	50
*assert*	2	25	5	3	35
*make clear*	2	27	1	4	34
*affirm*	1	18	0	3	22
*remark*	0	14	0	1	15
*contend*	1	9	1	2	13
*remind NOUN*	1	10	0	1	12
*intimate*	3	4	3	1	11
*inform NOUN*	3	6	1	0	10
*disclose*	1	5	1	1	8
*hypothesize*	0	3	0	0	3
*predict*	0	1	1	0	2
*divulge*	0	0	0	1	1
**Total**	**15118**	**6083**	**12804**	**3401**	**37406**

**Table B2.** Survey B: Predicates of belief, knowledge, and conjecture.

**Verb lemma**	**1st person subject**		**2nd person subject**		**Total**
	**Ø**	***that***	**Ø**	***that***	
*think*	36331	1800	20549	816	59496
*believe*	2723	578	2269	413	5983
*guess*	3623	34	1576	20	5253
*know*	1887	489	478	46	2900
*remember*	989	292	570	157	2008
*suppose*	682	83	1144	62	1971
*realize*	787	616	305	191	1899
*assume*	241	133	869	215	1458
*notice*	249	234	304	169	956
*forget*	514	172	173	77	936
*figure*	441	62	345	30	878
*find out*	330	129	204	118	781
*imagine*	137	98	308	80	623
*agree*	184	306	32	54	576
*recall*	48	73	90	11	222
*discover*	66	91	26	39	222
*presume*	10	8	87	22	127
*be aware*	13	75	4	16	108
*conclude*	12	44	6	23	85
*fancy*	19	8	10	4	41
*maintain*	7	15	8	2	32
*conjecture*	4	2	4	1	11
**Total**	**49297**	**5342**	**29361**	**2566**	**86566**
